# Achieving 2.2 GPa Ultra-High Strength in Low-Alloy Steel Using a Direct Quenching and Partitioning Process

**DOI:** 10.3390/ma16247533

**Published:** 2023-12-06

**Authors:** Gang Niu, Donghao Jin, Yong Wang, Haoxiu Chen, Na Gong, Huibin Wu

**Affiliations:** 1 Collaborative Innovation Center of Steel Technology, University of Science and Technology Beijing, Beijing 100083, China; ustbniug@163.com (G.N.); jindh_ustb@163.com (D.J.); 2 School of Materials Science and Engineering, Nanyang Technological University, Singapore 639798, Singapore; yong054@e.ntu.edu.sg; 3 Department of Materials Science and Engineering, University of Toronto, Toronto, ON M5S 3E4, Canada; haoxiu.chen@mail.utoronto.ca; 4 Institute of Materials Research and Engineering (IMRE), A⁎STAR (Agency for Science, Technology, and Research), 2 Fusionopolis Way, Singapore 138634, Singapore

**Keywords:** 2.2 GPa ultra-high strength steel, TMCP-DQP process, martensite, retained austenite, mechanical properties

## Abstract

Advanced high-strength steels (AHSS) have a wide range of applications in equipment safety and lightweight design, and enhancing the strength of AHSS to the ultra-high level of 2 GPa is currently a key focus. In this study, a new process of thermo-mechanical control process followed by direct quenching and partitioning (TMCP-DQP) was developed based on Fe-0.4C-1Mn-0.6Si (wt.%) low-alloy steel, and the effects of microstructure evolution on mechanical properties under TMCP-DQP process and conventional hot rolled quenched and tempered process (HR-QT) were comparatively studied. The results show that the TMCP-DQP process not only shortened the processing steps but also achieved outstanding comprehensive mechanical properties. The TMCP-DQP steel exhibited a tensile strength of 2.23 GPa, accompanied by 11.9% elongation and a Brinell hardness of 624 HBW, with an impact toughness of 28.5 J at −20 °C. In contrast, the HR-QT steel exhibited tensile strengths ranging from 2.16 GPa to 1.7 GPa and elongations between 5.2% and 12.2%. The microstructure of TMCP-DQP steel primarily consisted of lath martensite, containing thin-film retained austenite (RA), nanoscale rod-shaped carbides, and a minor number of nanoscale twins. The volume fraction of RA reached 7.7%, with an average carbon content of 7.1 at.% measured by three-dimensional atom probe tomography (3DAP). Compared with the HR-QT process, the TMCP-DQP process resulted in a finer microstructure, with a prior austenite grain (PAG) size of 11.91 μm, forming packets and blocks with widths of 5.12 μm and 1.63 μm. The TMCP-DQP process achieved the ultra-high strength of low-alloy steel through the synergistic effects of grain refinement, dislocation strengthening, and precipitation strengthening. The dynamic partitioning stage stabilized the RA through carbon enrichment, while the relaxation stage reduced a small portion of the dislocations generated by thermal deformation, and the self-tempering stage eliminated internal stresses, all guaranteeing considerable ductility and toughness. The TMCP-DQP process may offer a means for industries to streamline their manufacturing processes and provide a technological reference for producing 2.2 GPa grade AHSS.

## 1. Introduction

In recent years, the steel industry has been working towards the strategic goals of “carbon neutrality” and “peak carbon emissions”, making carbon emission reduction a shared priority [1]. This effort involves streamlining industrial production processes and adopting lightweight designs for products to minimize their carbon footprint [2]. Among the various types of steel, advanced high-strength steel (AHSS) stands out for its exceptional strength and plasticity, offering numerous applications to enhance equipment safety and achieve lightweight objectives [3]. Current materials research focuses on pushing the performance of AHSS to ultra-high strength levels, targeting 2 GPa. The realm of AHSS includes dual-phase steel (DP) [4], complex-phase steel (CP) [5], transformation-induced plasticity-aided steel (TRIP) [6], twinning-induced plasticity steels (TWIP) [7], austenitic stainless steels (ASS) [8], quenching and partitioning processed steel (Q&P) [9], and carbide-free bainitic (CFB) steel [10]. In 2003, Speer et al. [11] introduced the quenching and partitioning (Q&P) process, where steel is quenched and cooled within the martensite transformation temperature range after partial or full austenitization. This process facilitates the diffusion of carbon atoms from oversaturated martensite to untransformed austenite, thereby enriching the carbon content in the austenite. The outcome is a microstructure composed of martensite and well-stabilized retained austenite (RA) at room temperature. The martensite contributes to a higher ultimate tensile strength (UTS, 800–1500 MPa), while the TRIP effect enabled by RA leads to a significant increase in total elongation (TE, 10.0–30.0%) [12,13].

The mechanical properties of Q&P steel are intricately tied to several key factors, including the volume fraction, size, morphology, and distribution of its constituent phases, as well as precise control over heat treatment parameters. Over time, researchers have sought to optimize the Q&P heat treatment process parameters to enhance the overall mechanical performance of the steel. For example, by fine-tuning the quenching temperature, the initial volume fractions of martensite and untransformed austenite can be accurately determined [14]. Moreover, elevating the quenching temperature has the added effect of promoting the formation of blocky RA [15]. The choice of partitioning temperature allows for manipulation of the extent of martensite transformation. The combined impact of partitioning temperature and partitioning time plays a crucial role in the carbon partitioning process, thereby influencing the stability of RA [16,17]. Additionally, some researchers have explored the influence of heating rate on the microstructural evolution and mechanical properties of Q&P steel [18]. Ultra-fast heating can delay the recrystallization of deformed ferrite and increase the nucleation rate of austenite, resulting in a finer austenite grain size. As evidenced by the results, subjecting Q&P steel to ultra-rapid heating (at a rate of 300 °C/s) yields a significant strength boost, raising it from 980 MPa to 1180 MPa compared with conventionally heated Q&P steel.

Traditional Q&P treatments have conventionally been carried out on offline heat treatment production lines subsequent to hot rolling. This method entails high energy consumption and often results in low production efficiency. Responding to the growing need for more streamlined and efficient production processes, an increasing number of researchers are now directing their focus towards post-hot rolling direct quenching and partitioning (DQP) processes [19]. In contrast to the traditional Q&P approach, DQP treatment involves a dynamic partitioning process. It leverages the residual heat from hot-rolled steel plates for partitioning, effectively reducing the energy consumption associated with repeated reheating cycles. Furthermore, the DQP process offers several advantages, including the ability to attain the desired microstructure and excellent mechanical properties [19,20,21,22]. For instance, Tan et al. [20] applied DQP and isothermal partitioning processes to low-carbon steel, investigating the distinctions in microstructure and mechanical properties under these various treatments. The findings revealed that, in comparison with isothermal partitioning, DQP samples exhibited narrower martensite laths and higher dislocation densities. These characteristics became more pronounced with an increase in the cooling rate during the dynamic partitioning process, resulting in higher tensile strength (1300–1600 MPa) and similar elongation (14–18%). In a separate study, Parthiban et al. [21] compared the mechanical properties of cast steel subjected to quenching and partitioning treatment, DQP treatment following thermo-mechanical controlled processing (TMCP), and offline quenching and partitioning treatment (SQP) after TMCP. The results demonstrated that the tensile strength and elongation changed from 882 MPa and 18.5% for cast steel to 1398 MPa and 11% for TMCP-DQP treatment, and 1327 MPa and 13% for TMCP-SQP treatment. This illustrates that the high-density dislocations and fine grains produced through the TMCP process can significantly enhance the hardening ability of steel without notably affecting its elongation.

While the current AHSS produced through the DQP process exhibits impressive mechanical properties, surpassing the 2 GPa tensile strength threshold remains a formidable challenge. A comprehensive examination of the synergistic effects of TMCP technology and dynamic partitioning processes on the mechanical properties of steel, as well as the underlying microstructure evolution mechanisms, is currently lacking. Therefore, building upon prior investigations [23,24,25], this study aimed at developing an AHSS with a tensile strength of 2.2 GPa, while preserving exceptional plasticity and toughness. Compared with traditional hot-rolled quenching and tempering (HR-QT), this objective will be accomplished by a process that integrates thermo-mechanical rolling with direct quenching and partitioning (TMCP-DQP). Multiple characterization techniques will be employed to examine phase compositions, martensitic structural characteristics, RA volume fractions, and internal carbon content. Furthermore, the study will dissect the microstructure evolution mechanisms of steel during the TMCP-DQP process.

## 2. Experimental Materials and Methods

### 2.1. Material Processing

**Figure 1:**
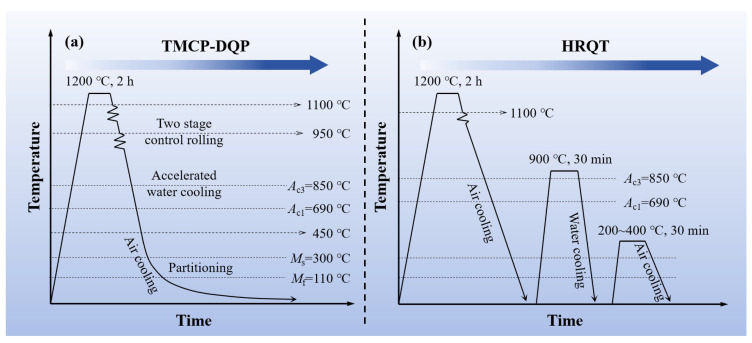
Heat treatment process of the studied steel. (**a**) TMCP-DQP; (**b**) HR-QT.

**Figure 2:**
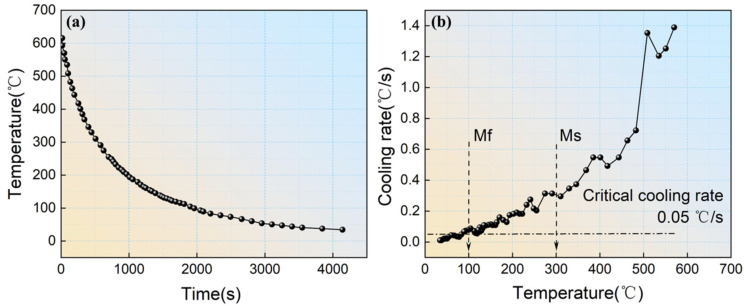
Temperature versus time curve (**a**) and differential curve (**b**) during the air cooling stage in the TMCP-DQP process.

The steel investigated in this study was Fe–0.4C–1Mn–0.6Si (wt.%) alloy manufactured via vacuum induction melting (Shenyang Jinyan Co., Ltd., Shenyang, China). Detailed information regarding its chemical composition can be found in Table 1. The original ingot was hot forged into billets with dimensions of 60 mm × 60 mm (width × thickness). These billets underwent a homogenization process, holding at 1200 °C for 12 h. Critical temperatures were determined with a Bähr DIL805A dilatometer delivered by TA Instruments (Zaventem, Belgium), yielding the following results: austenite start temperature (AC_1_) = 690 °C, austenite finish temperature (AC_3_) = 850 °C, martensite start temperature (M_s_) = 300 °C, and martensite finish temperature (M_f_) = 110 °C. The critical cooling rate for martensite transformation, as derived from the continuous cooling transformation (CCT) curve, was approximately 0.05 °C/s. Two distinct production processes were employed for the steel billets. The first process, as depicted in Figure 1a, was the TMCP-DQP process. It involved the following steps: heating the steel billets to 1200 °C and holding them for 2 h, with subsequent two-stage rolling starting from 1100 °C with a total of three passes until the steel ingots reached a thickness of 12 mm; water cooling took place after rolling until the surface temperature reached 450 °C, followed by air cooling to room temperature. The resulting samples from this process were named TMCP-DQP. Temperature changes during the air cooling process after rolling were monitored using a precision thermocouple with 1 °C accuracy, as illustrated in Figure 2a. The derivative of the curve showed the cooling rate at different temperatures, as demonstrated in Figure 2b. Since the cooling rates between M_s_ and M_f_ were all higher than 0.05 °C/s and the cooling process was relatively slow (20 min), this cooling process in the TMCP-DQP process was considered a dynamic partitioning process. The second process, as shown in Figure 1b, was the HR-QT process. It involved the following steps: initial heating of the steel billets to 1100 °C, followed by conventional hot rolling to a final thickness of 12 mm; air cooling to room temperature; subsequent reheating of the plates to 900 °C for 30 min to re-austenitize, followed by water quenching; tempering at various temperatures (200, 250, 300, 350, and 400 °C) for 30 min each. The resulting samples from this process were named HR-QT200, HR-QT250, HR-QT300, HR-QT350, and HR-QT400, depending on the tempering temperature.

### 2.2. Microstructure Characterization

The microstructural characteristics of both types of steel were examined using metallographic samples. Sample preparation involved mechanical grinding, polishing, and etching with a 4% nitric acid alcohol solution. Subsequently, these prepared samples were analyzed using a scanning electron microscope (SEM, Gemini SEM 500, Zeiss, Jena, Germany). After regrinding and polishing, the samples were immersed in the prior austenite grain boundaries’ (PAGBs) etchant (picric acid solution) and etched for 6–8 min at a constant temperature in a 70 °C water bath. The prior austenite grain (PAG) size was then determined using an optical microscope (OM). For further analysis, the samples were subjected to electrochemical polishing in a 10% perchloric acid electrolyte solution to facilitate electron backscattering diffraction (EBSD) analysis, with a spatial step size of 0.015 µm. To prepare thin foils for transmission electron microscopy (TEM), 3 mm-thick foils obtained through wire cutting were mechanically polished down to a thickness of 70 μm. Electrolytic polishing was performed using a DJ2000 twin-jet electropolisher (Hanyu, Beijing, China) in a 5% perchloric acid alcohol solution at −30 °C. Additionally, the fine structure and elemental distribution of the samples were investigated using a transmission electron microscope (TEM, Tecnai G2 F20, FEI, Hillsboro, OR, USA). Energy-dispersive spectroscopy (EDS) was used in conjunction to provide elemental analysis operating under 200 kV conditions. To further understand the atomic arrangement, three-dimensional atom probe tomography (3DAP) experiments were conducted using Cameca LEAP 4000XP equipment (Cameca, Fitchburg, WI, USA)in voltage pulsing mode. The experiments were carried out while maintaining a temperature of 60–70 K, a pulse rate of 200 kHz, and a pulse fraction of 20%.

X-ray diffraction (XRD) measurements were performed using a DMAX-RB 12 kW rotating anode diffractometer (Rigaku, Tokyo, Japan) operating at 40 kV and 150 mA. The spectrum was scanned in the 2θ range from 40° to 120° at a scanning rate of 0.30°/min. Prior to the XRD testing, the samples underwent electrolytic polishing to minimize errors arising from residual stresses introduced during sample preparation. At room temperature, Cu-Kα radiation was employed to determine the RA content and average carbon concentration. The intensities of the (200)α, (211)α, (200)γ, (220)γ, and (311)γ peaks were integrated, and the RA volume fraction was calculated using the following formula [26]:

(1) $$V_{\gamma }=1.4I_{\gamma }/\left(I_{\alpha }+1.4I_{\gamma }\right)$$

where $V_{\gamma }$ represents the volume fraction of RA; $I_{\gamma }$ and $I_{\alpha }$ represent the average integrated intensities of the austenite and ferrite peaks, respectively.

The formula for calculating the average carbon concentration of RA is as follows [27]:

(2) $$C_{\gamma }=\left(\alpha_{\gamma }-3.547\right)/0.046$$

where $C_{\gamma }$ represents the weight percentage of carbon concentration in austenite; $\alpha_{\gamma }$ stands for the lattice parameter of the austenite phase, which can be calculated based on the average values of the (200), (220), and (311) austenite peaks using the following formula:

(3) $$\alpha_{\gamma }=\frac{\lambda \sqrt{h^{2}+k^{2}+l^{2}}}{2sin\theta_{hkl}}$$

where λ represents the wavelength of the radiation; $\left(hkl\right)$ refers to the three Miller indices of a crystallographic plane; $\theta_{hkl}$ is the Bragg angle.

### 2.3. Mechanical Performance Tests

Tensile and Charpy V-notch impact tests were carried out on samples, cut longitudinally along the rolling direction of the steel plate. The tensile specimens were characterized by a gauge length of 25 mm, a width of 5 min, and a thickness of 1.5 mm. Uniaxial tensile tests were performed using an HTM 16,020 tensile testing machine (ZwickRoell GmbH & Co. KG, Ulm, Germany), operating at a testing speed of 1 mm/min. Charpy V-notch impact test specimens were of dimensions 10 mm × 10 mm × 50 mm and were tested at −20 °C utilizing a JBW-450 oscilloscope impact toughness tester (Fangyuan, Jinan, China) to evaluate the impact energy for both processes. A minimum of three samples for each process were subjected to tensile and impact tests, and the averages were computed to ensure data consistency. The Brinell hardness values of the samples were determined using an HBS-3000 Brinell hardness tester (Fangyuan, Jinan, China) with a testing force of 187.5 kg. For each of the two steel types, a minimum of 20 hardness measurements were taken, and the average was calculated after excluding the maximum and minimum values to enhance accuracy.

## 3. Results

### 3.1. Mechanical Properties

**Figure 3:**
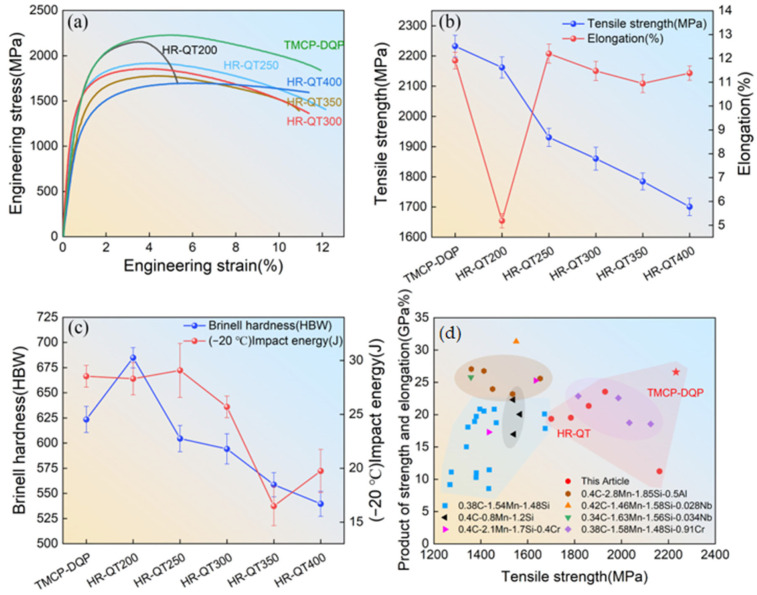
Mechanical performance of TMCP-DQP steel and HR-QT steel at different tempering temperatures. (**a**) Engineering stress–strain curves, (**b**) tensile strength and elongation, (**c**) Brinell hardness and impact energy, and (**d**) comparison of TMCP-DQP steel to other steels with similar C (0.4 wt.%) content in terms of production of strength and elongation versus tensile strength.

Figure 3 illustrates the mechanical performance of TMCP-DQP steel and HR-QT steel at various tempering temperatures. HR-QT steel displayed a tensile strength ranging from 2162 MPa to 1700 MPa, coupled with an elongation varying between 5.2% and 12.2%. The Brinell hardness and impact energy spanned from 539.6 HB to 684.8 HB and 16.5 J to 29.1 J, respectively. In contrast, TMCP-DQP steel exhibited exceptional mechanical properties, boasting the highest tensile strength at 2233 MPa. It also achieved an elongation of 11.9%, only slightly lower than HR-QT250 (12.2%). The Brinell hardness of TMCP-DQP steel was 623.5 HB, marginally lower than HR-QT200 (684.8 HB). Furthermore, TMCP-DQP steel demonstrated impressive impact performance, reaching 28.5 J, which closely matched HR-QT250 (29 J). It is worth noting that the tensile strength and Brinell hardness of HR-QT steel decreased as the tempering temperature increased. HR-QT200 exhibited only 5.2% elongation, indicating that intense quenching brittleness remained after tempering at 200 °C. However, when tempering temperatures exceeded 250 °C, HR-QT steel showed elongations exceeding 10%. The significant change in elongation may be closely related to the disappearance of residual stress and quenching brittleness. Notably, with the tempering temperature rising to 350 °C, the elongation slightly decreased, and the impact performance significantly dropped, indicating mild temper brittleness, with impact energy as low as 16.5 J at −20 °C. In summary, TMCP-DQP steel demonstrated superior strength and ductility. In comparison with other Q&P steels with a similar carbon content (0.4 wt.% C) from the existing literature [28,29,30,31,32,33,34], TMCP-DQP steel achieved a substantial increase in tensile strength and exhibited leading strength–ductility combinations.

### 3.2. Microstructure Characterization

**Figure 4:**
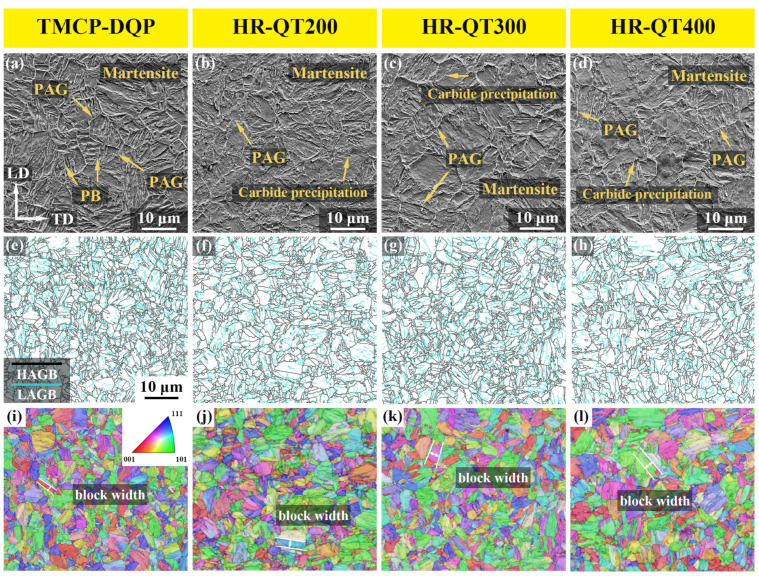
Microstructures of TMCP-DQP steel and HR-QT steel. (**a**) SEM image of TMCP-DQP steel; (**b**–**d**) SEM images of HR-QT200, HR-QT300, and HR-QT400 steels; (**e**) LAGBs and HAGBs in TMCP-DQP steel; (**f**–**h**) LAGBs and HAGBs in HR-QT200, HR-QT300, and HR-QT400 steels (LAGBs between 2° and 15° are represented by blue lines, while HAGBs with orientation differences greater than 15° are represented by black lines); (**i**) EBSD inverse pole figure (IPF) map of TMCP-DQP steel; (**j**–**l**) EBSD inverse pole figure (IPF) map of HR-QT200, HR-QT300, and HR-QT400 steels.

**Figure 5:**
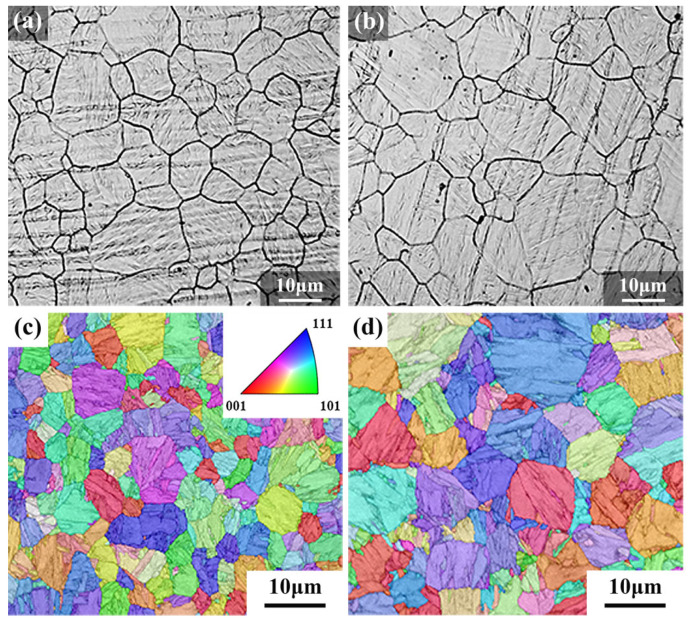
Prior austenite grain images of TMCP-DQP steel and HR-QT steel. (**a**,**b**) prior austenite grain boundaries obtained by the etching method for TMCP-DQP steel and HR-QT400 steel; (**c**,**d**) prior austenite grain images obtained by EBSD data reverse reconstruction method for TMCP-DQP steel and HR-QT400 steel.

Figure 4 displays the microstructures of TMCP-DQP steel and HR-QT steel. From the observation of SEM microstructures, both exhibited tempered martensite, with clearly visible PAGBs. Notably, in TMCP-DQP steel, the martensite laths featured prominent boundaries (Figure 4a), facilitating statistical analysis. In HR-QT steel, as the tempering temperature rose, the number of precipitated carbides gradually increased (Figure 4b–d). Martensite lath colonies consisted of laths with the same crystal orientation, while low-angle grain boundaries (LAGBs) mainly comprised dislocations and sub-blocks. High-angle grain boundaries (HAGBs) included PAGBs, martensite lath colony boundaries, and lath block boundaries [35]. Combining grain boundary distribution in Figure 4e–h and orientation distribution in Figure 4i–l enabled the identification of lath widths in TMCP-DQP steel and HR-QT steel. Figure 5 showcases the reconstructed images of the PAG in TMCP-DQP steel and HR-QT400 steel. The reconstruction method of PAG was crucial for statistically analyzing grain sizes. To ensure accuracy, this study used two methods: solution etching (Figure 5a,b) and EBSD data reconstruction methods (Figure 5c,d). AZtecCrystal 2.1.2 software was employed for the reconstruction, involving denoising and reverse reconstruction of the PAG orientations in TMCP-DQP steel (Figure 4i) and HR-QT400 steel (Figure 4l) based on the Kurdjumov–Sachs orientation relationship (K-S relationship). As the production process for HR-QT steel solely differed in tempering temperature, it did not affect the PAG size. Therefore, the PAG size for HR-QT steel was represented by HR-QT400 steel. The results revealed that TMCP-DQP steel featured finer microstructures and substructures, with approximately 11.91 ± 0.61 μm for the PAG size, 5.12 ± 0.21 μm for the martensite lath colony width (packet), and 1.63 ± 0.06 μm for the martensite lath block width (block). In contrast, HR-QT steel possessed a PAG size of about 15.05 ± 0.47 μm, and its microstructural substructure merged with increasing tempering temperature. The widths of the packet and block increased from 5.63 ± 0.18 μm to 7.47 ± 0.16 μm and from 2.37 ± 0.08 μm to 3.02 ± 0.05 μm, respectively. Statistical results are summarized in Table 2.

**Figure 6:**
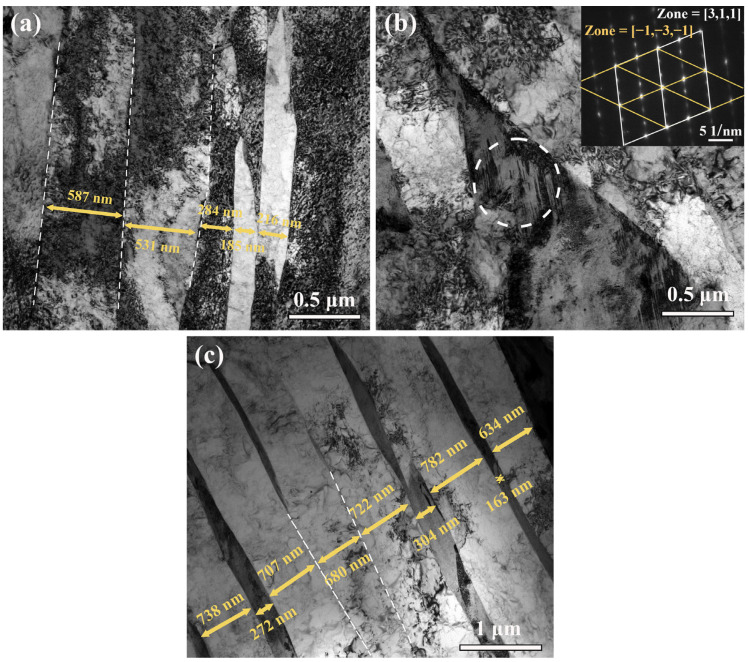
TEM images of TMCP-DQP steel and HR-QT steel. (**a**) Martensite laths in TMCP-DQP steel, (**b**) twin structure (white circle) and diffraction pattern in TMCP-DQP steel, and (**c**) martensite laths in HR-QT300 steel.

Figure 6 shows TEM images of the two types of steel. The martensite laths in both steels were nearly parallel and had clear lath boundaries (Figure 6a,c). HR-QT300 steel laths contained fewer dislocations; while, in contrast, many dislocations and entanglements were observed in the TMCP-DQP steel laths. Statistical analysis indicated an average martensite lath width of 363.5 nm for TMCP-DQP steel, which was smaller than the average lath width of 515.8 nm for HR-QT300 steel. Since the experimental steel contained a higher carbon content, local intragranular twin structures that did not penetrate the entire martensite laths could be observed in TMCP-DQP steel (Figure 6b). These twin structures were oriented at an approximately 45 °C angle to the martensite lath direction, and their diffraction patterns exhibited distinct twinning features.

**Figure 7:**
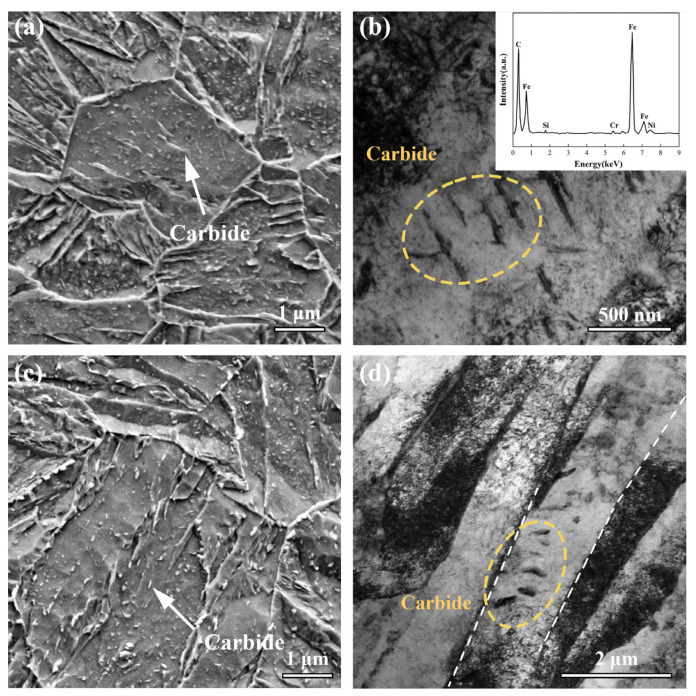
Carbide morphology in TMCP-DQP steel and HR-QT steel. (**a**) SEM image of carbides in HR-QT300 steel, (**b**) TEM images of carbides in TMCP-DQP steel along with corresponding EDS analysis, (**c**) SEM image of carbides in HR-QT300 steel, and (**d**) TEM images of carbides in HR-QT300 steel.

By examining the microstructure at high magnification through SEM and TEM (in conjunction with EDS analysis), Figure 7 illustrates the morphology of observed precipitates. These precipitates, displaying a specific orientation relationship with the matrix, were identified as carbides. In TMCP-DQP steel, the carbides manifested rod-like and elliptical shapes. The rod-like carbides exhibited a thickness ranging from approximately 25 to 50 nm (Figure 7b), while elliptical carbides measured about 15–30 nm. In HR-QT300 steel, where tempering was prolonged, rod-like carbides progressively transformed into elliptical shapes. Furthermore, elliptical carbides underwent coarsening, resulting in an average size of 25–75 nm (Figure 7d).

**Figure 8:**
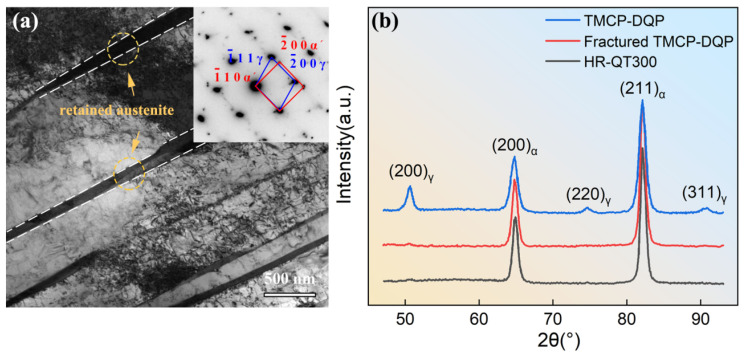
Morphology of retained austenite in both TMCP-DQP steel and HR-QT300 steel. (**a**) TEM morphology of retained austenite in TMCP-DQP steel; (**b**) XRD results of TMCP-DQP steel before and after tensile testing, as well as HR-QT300 steel.

Figure 8a displays the morphology of RA in TMCP-DQP steel, with electron diffraction patterns confirming its presence between martensite laths in a thin film-like form. In Figure 8b, XRD patterns of TMCP-DQP steel, the tensile fracture of TMCP-DQP steel, and HR-QT300 steel are presented. Calculations based on the patterns revealed that the volume fraction of RA in TMCP-DQP steel reached 7.7 vol%, with an average carbon concentration of 1.43 wt.% (6.3 at%). This carbon concentration was higher than that of RA in steels with similar carbon content (0.4 wt.%), indicating that the TMCP-DQP process effectively promoted the carbon element partitioning. XRD analysis of TMCP-DQP steel after fracture showed that nearly all RA transformed into strain-induced martensite. In HR-QT200 steel, the volume fraction of RA was below the lower limit of the detection capability of the instrument, measuring just 3%, which can be neglected.

## 4. Discussion

**Figure 9:**
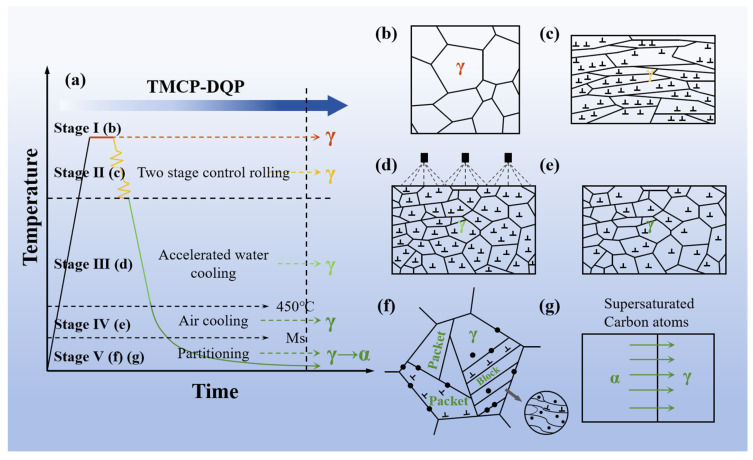
Microstructural evolution schematic of TMCP-DQP steel. (**a**) TMCP-DQP process, (**b**) austenitization microstructures, (**c**) post-TMCP microstructures, (**d**) microstructures during rapid water cooling, (**e**) microstructures during the relaxation phase, (**f**) carbon partitioning schematic, and (**g**) martensite structure schematic.

Compared with HR-QT steel, the outstanding mechanical performance of TMCP-DQP steel was closely related to its microstructural evolution, as illustrated in Figure 9. In the first stage of the austenitization process (Figure 9b), an appropriate holding time ensured a uniform element distribution while preventing excessive grain coarsening of the austenite grains, which could lead to a deterioration in mechanical performance. Subsequently, during the two-stage controlled rolling process (Figure 9c), the grains were elongated and refined, and the dislocation density generated by intragranular deformation increased significantly. Due to the higher temperature, dynamic recrystallization was evident, ensuring that the PAG remained fine when cooling to M_s_ (300 °C), as depicted in Figure 5. This fine state served as the foundation for the formation of a refined substructure during martensitic transformation. Immediately after the controlled rolling, accelerated water cooling was applied (Figure 9d). This limited the growth of over-saturated and deformation-induced carbides during the cooling process and restricted the excessive growth of grains and subgrain coarsening that could occur after the second-stage rolling. This was advantageous for obtaining fine martensite laths, which contributed significantly to the strength and toughness of the material. During the air-cooling process from the cooling endpoint temperature (450 °C) to M_s_ (300 °C), static recovery occurred within the subcooled austenite (Figure 9e). During this phase, dislocations generated by deformation were rearranged, leading to a reduction in dislocation density and the elimination or reduction of residual stresses. This helped avoid plastic damage that could occur when a large number of transformation dislocations coexisted with deformation-induced dislocations during the martensitic transformation. As the temperature decreased to M_s_, the measured air cooling rate of the experimental steel was 0.3 °C/s (Figure 2b), which was higher than the critical cooling rate of 0.05 °C/s required for martensitic transformation, leading to the formation of martensite (Figure 9f). During this transformation phase, on the one hand, the martensitic transformation process was relatively “slow”. On the other hand, the remaining austenite that had not yet transformed continued to experience carbon atom diffusion partitioning with the newly transformed martensite (Figure 9g). The partitioning time was sufficient to stabilize the untransformed austenite. This mechanism was similar to the one-step Q&P process. Finally, as the temperature dropped below M_f_, high-carbon RA with a volume fraction of 7.7 vol% and a multitude of lath martensite was achieved. The RA existed as thin films within the fine gaps between the laths (Figure 8a).

The stability of RA was crucial for the mechanical properties of ultra-high strength steels. The most effective method to enhance the stability of RA was to promote carbon enrichment. Additionally, the size and morphology of austenite also played a crucial role in this process [36,37,38]. To visually characterize the morphology of RA in TMCP-DQP steel and detect its enriched carbon concentration, a three-dimensional atom probe (3DAP) layer analysis was employed. Integrated visualization and analysis software (IVAS) was used to analyze the elemental distribution in the obtained regions. The “equal concentration surface” method was used to mark regions with a specified carbon concentration value, thus forming an equal concentration surface. In this case, a carbon concentration threshold value of 4.0 at.% was defined as the boundary for the RA regions, and equal concentration surface analysis parameters of 4.0, 6.0, and 8.0 at.% carbon concentrations were chosen to obtain the three-dimensional distribution of carbon-enriched microregions in the steel, as shown in Figure 9a. It could be observed that the shape of the RA regions defined by the 4.0 at.% equal concentration surface was primarily film-like, with some areas containing smaller highly carbon-enriched block-like regions. As the equal concentration surface gradually increased to 6.0 and 8.0 at.%, the RA regions enclosed by the equal concentration surface slightly reduced in size, with an increased proportion of block-like regions. This indicated that the distribution of carbon elements inside the entire RA region was not entirely uniform, and there existed a transition layer where the carbon concentration gradually increased towards the interior.

**Figure 10:**
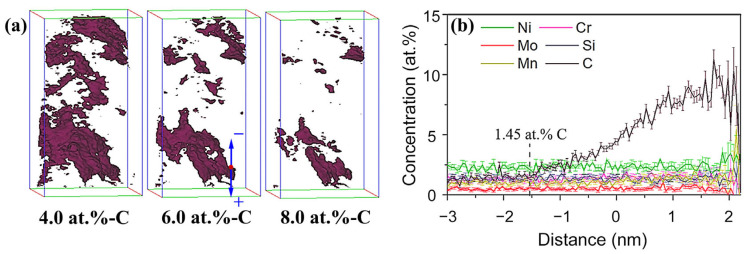
Retained austenite in TMCP-DQP steel. (**a**) Three-dimensional distribution of retained austenite at different carbon equal concentration surfaces; (**b**) distribution of the major alloying elements at various distances from the 6.0 at.% carbon equal concentration surface.

Composition analysis was performed on the RA in Figure 10a. A carbon concentration of 6.0 at.% was selected as the reference plane, with the position representing a statistical concentration of carbon at 6.0 at.% marked as 0 nm. The concentration distribution of the major alloying elements at different distances from the reference plane is shown in Figure 10b. It was obvious that the carbon concentration varied significantly with position, with a transition region of only 2 nm from enrichment to the equal concentration surface, while the concentrations of other elements remained nearly constant with position. As the sampled region used for concentration statistics became smaller in higher concentration regions, there was a relatively larger concentration error displayed in the graph. The average carbon content in the RA phase reached 7.1 at.%, which closely matched the XRD results. In stark contrast, the carbon content in the martensite phase was slightly lower than the average carbon content of the experimental steel, as shown in Table 3. Comparing the content of other alloying elements in the RA and martensite regions, it was evident that only carbon atoms underwent partitioning during the dynamic partitioning process.

The diffusion of carbon during the partitioning process was highly sensitive to both time and temperature [39]. Short-duration partitioning treatments, typically lasting a few tens of seconds, facilitated carbon diffusion from martensite to RA, leading to carbon enrichment in the RA and enhancing its stability. The difference in carbon chemical potential between the two phases provided the driving force for carbon partitioning. On the other hand, in the TMCP-DQP process, longer partitioning times could lead to the formation of carbides due to the enrichment of carbon diffusion, as shown in Figure 7. Therefore, during the self-tempering process of martensite, there was a competitive relationship between effective carbon partitioning and carbide formation processes [40].

**Figure 11:**
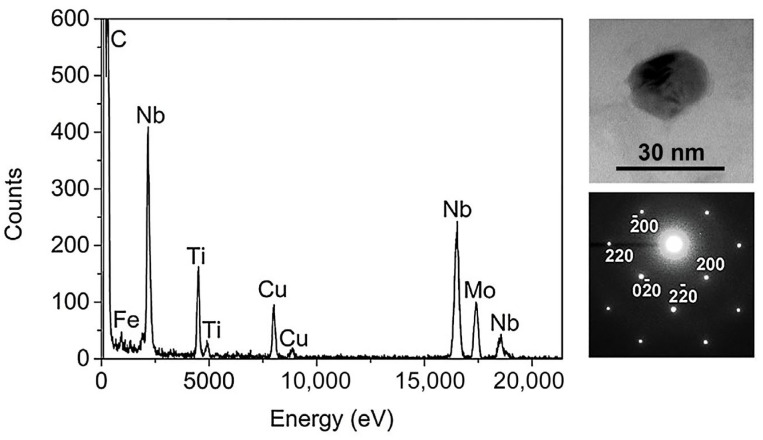
Typical morphology of the precipitate particles in TMCP-DQP steel along with their corresponding EDX spectra and electron diffraction patterns.

To observe the carbide particles in TMCP-DQP steel in greater detail, representative carbides were obtained using an extraction and replication technique. TEM images and selected area electron diffraction (SAED) patterns of these carbide particles are shown in Figure 11. The carbide particles had sizes ranging from approximately 15 to 30 nm. The chemical composition of these precipitates was analyzed through energy-dispersive X-ray (EDX) spectroscopy. The results of the EDX analysis revealed that these precipitate particles within the TMCP-DQP steel contained not only Nb and Ti elements but also small amounts of Mo, indicating that they were carbides containing Nb, Ti, and Mo. Quantitative EDX analysis results showed the Nb/Ti and Ti/Mo elemental ratios within these particles to be about 2.99 ± 0.60 and 2.09 ± 0.84, respectively. According to the corresponding diffraction patterns in Figure 10, these carbide particles exhibited a NaCl-type crystal lattice structure, confirming that they were MC-type carbides [41].

The ultra-high strength exceeding 2 GPa in TMCP-DQP steel presented in this study could be attributed to several critical factors. First, it benefited from the controlled rolling process and the dynamic recrystallization that led to the formation of fine austenite during the phase transformation process. This resulted in the development of a fine substructure (packet and block structures, as illustrated in Table 1) that initiated grain refinement strengthening. Additionally, the presence of fine martensite platelets in the microstructure, along with dislocations generated through deformation and phase transformation, contributed to further strengthening. Moreover, during the self-tempering process between the M_s_ and M_f_ temperatures in TMCP-DQP steel, nanoscale carbides precipitated, providing an additional strengthening effect. In contrast, HR-QT steel experienced a re-austenitization process, leading to some coarsening of the PAG and microstructures. This process resulted in wider martensite platelets. The variation in tempering degrees affected the density of phase transformation dislocations and the growth of carbide particles, ultimately leading to a reduction in strength. Furthermore, the excellent ductility of TMCP-DQP steel benefited from the TRIP effect, achieved through the dynamic carbon partitioning process during the self-tempering phase between M_s_ and M_f_ temperatures. This effect effectively alleviated stress concentration, promoted strain hardening, and delayed necking [42,43,44]. Additionally, the relaxation phase before martensitic transformation eliminated a portion of the dislocations. During the martensite transformation, the slow cooling process led to low-temperature self-tempering, which, to some extent, helped eliminate internal stresses. In contrast, HR-QT steel lacked stable RA and necessitated an increase in tempering temperature to approximately 250 °C to eliminate quenching brittleness and achieve equivalent ductility and impact resistance to TMCP-DQP steel. However, this inevitably resulted in a loss of strength, making it challenging to achieve both ultra-high strength and excellent ductility simultaneously.

## 5. Conclusions

(1) The Fe-0.4C-1Mn-0.6Si (wt.%) steel, processed through the TMCP-DQP technique, demonstrated excellent mechanical properties while significantly shortening the manufacturing process. It achieved a tensile strength of 2233 MPa, accompanied by an elongation of 11.9%, a hardness of 624 HBW, and an impact energy of 28.5 J at −20 °C. In contrast, steel treated by HR-QT processing exhibited a wide range of tensile strengths, from 2162 MPa to 1700 MPa, and elongation ranging from 5.2% to 12.2%. The Brinell hardness values and impact energies spanned from 539.6 HB to 684.8 HB and 16.5 J to 29.1 J, respectively. Both tensile strength and hardness decreased as tempering temperature rose, whereas elongation and impact toughness initially increased and then decreased with increasing tempering temperature. Mild temper embrittlement was observed at tempering temperatures greater than 250 °C.

(2) TMCP-DQP steel exhibited a microstructure dominated by lath martensite, including RA in a film-like form, nanoscale rod-shaped carbides, and a small number of fine twin boundaries. The average width of the martensite laths was 363.5 nm, with a substantial presence of dislocation tangles on the lath boundaries. The relative content of RA reached 7.7 vol.%, with an average carbon content of 7.1 at.%. Compared with HR-QT steel, the TMCP-DQP process resulted in a finer microstructure and substructure. The PAG size was 11.91 μm, subsequently forming packet and block structures with widths of 5.12 μm and 1.63 μm during the subsequent phase transformation.

(3) The ultra-high strength of TMCP-DQP steel was partly attributed to grain refinement caused by fine austenite formed during controlled rolling and dynamic recrystallization and fine martensite laths generated in the subsequent martensitic transformation. Furthermore, the nanoscale carbides and dislocations generated by deformation and phase transformation provided additional strengthening to the TMCP-DQP steel. The good ductility and toughness of TMCP-DQP steel resulted from the TRIP effect induced by the stable RA obtained through the dynamic carbon partitioning process during the air-cooling stage. Additionally, the relaxation stage before martensite transformation eliminated a portion of the dislocations generated from deformation, and the low-temperature self-tempering during slow cooling of the final stage to some extent alleviated internal stresses. These combined factors ensured that TMCP-DQP steel not only achieved an ultra-high strength of 2.2 GPa but also exhibited outstanding ductility and toughness.

## Figures and Tables

**Table 1:** Dominant chemical compositions of the studied steel (wt.%).

Elements	C	Si	Mn	Cr	Ni	Mo	Nb	Ti	Fe
wt.%	0.40	0.61	1.06	1.20	2.05	0.54	0.062	0.016	Bal.

**Table 2:** Microstructure and substructure size statistics for TMCP-DQP steel and HR-QT steel.

	Prior Austenite Grain Size/μm	Packet Width/μm	Block Width/μm
TMCP-DQP	11.91 ± 0.61	5.12 ± 0.21	1.63 ± 0.06
HR-QT200	15.05 ± 0.47	5.63 ± 0.18	2.37 ± 0.08
HR-QT300	15.05 ± 0.47	6.26 ± 0.16	2.73 ± 0.08
HR-QT400	15.05 ± 0.47	7.47 ± 0.16	3.02 ± 0.05

**Table 3:** Content of some elements in both the martensite and retained austenite phases (at.%).

Phase	C	Si	Mn	Cr	Ni	Mo
M	1.45	1.36	1.02	1.24	2.25	0.27
RA	7.1	1.38	1.16	1.39	2.34	0.32

## Data Availability

Data are contained within the article.
